# Evaluation of blood and tooth element status in asthma cases: a preliminary case–control study

**DOI:** 10.1186/s12890-021-01565-9

**Published:** 2021-06-15

**Authors:** S. Songül Yalçın, Nagehan Emiralioğlu, Suzan Yalçın

**Affiliations:** 1 Department of Pediatrics, Faculty of Medicine, Hacettepe University, Ankara, Turkey; 2 Department of Food Hygiene and Technology, Faculty of Veterinary Medicine, Selçuk University, Konya, Turkey

**Keywords:** Asthma, Magnesium, Zinc, Mercury, Teeth, Blood

## Abstract

**Background:**

Asthma is a common respiratory disorder; some data were present on the correlation between increased levels of trace elements and the risk of asthma development. It was aimed to evaluate the levels of 13 selected blood and tooth elements (magnesium, phosphorus, calcium, chromium, manganese, iron, copper, zinc, strontium, molybdenum, cadmium, lead, mercury) in a well-controlled asthma group and the control group.

**Methods:**

During the study period, 17 asthma patients and 26 age and gender-matched healthy children donated shed deciduous teeth having neither decay nor filling and enrolled for the study. The element levels in blood and teeth matrixes were analyzed with inductively coupled plasma mass spectrometry. Differences in blood and tooth elements in groups were evaluated with generalized linear models after adjusting confounding factors.

**Results:**

After adjusting the child’s “z scores of body mass index for age”, history of iron deficiency anemia, and status of parental smoking, the generalized linear model revealed significantly lower tooth magnesium levels, lower blood zinc levels, and lower blood zinc/copper ratio in the asthma group than the control group (*p* = 0.042, *p* = 0.034, *p* = 0.002, respectively). Other studied elements for tooth and blood matrixes were similar in groups.

**Conclusion:**

Our study revealed some differences in tooth and blood element levels in the asthma group. Further studies on zinc and magnesium levels of severe asthma cases are necessary for the interpretation of the results.

**Supplementary Information:**

The online version contains supplementary material available at 10.1186/s12890-021-01565-9.

## Introduction

Asthma is a common chronic respiratory disorder with airway inflammation, and the prevalence of asthma has increased in the last years. It is supposed that the increase in the prevalence of asthma is also related to the additional effects of environmental factors and lifestyle changes in addition to the genetic tendency [1–4]. Elements such as calcium (Ca), phosphorus (P), iron (Fe), zinc (Zn), magnesium (Mg), copper (Cu), cobalt (Co), chromium (Cr), manganese (Mn), molybdenum (Mo), nickel (Ni) are important components for various biochemical and physiological functions of the human body. However, metals such as cadmium (Cd), lead (Pb), and mercury (Hg) threaten human health [2, 3, 5].

The main reason for asthma is a shift in the T helper 1 (Th1)/T helper 2 (Th2) balance towards a Th2 response leading to inflammation, and the risk of asthma development due to dysregulation of these metal homeostasis has been claimed recently [1, 2, 5]. This chronic inflammation in childhood asthma is triggered when the environmental factors lead to free oxidant radical production in genetically predisposed individuals. Furthermore, the concentration of these elements in serum may impact the antioxidant system, inflammation and airway hyper-responsiveness [6–8]. In studies using blood and urine samples, a cause–effect relationship cannot be established, only information about that moment can be obtained. Associations among cord blood, newborn hair, and breast milk were shown [9]. However, there is no study for interaction between maternal blood samples during the antenatal period and teeth samples of the child. Whereas, shed baby dental samples were analyzed in congenital heart diseases, obesity, and neurodevelopmental disorders to provide information about the prior element status, intrauterine, and infancy period [10–12]. After the development of baby teeth at the embryonic stage of the fetus, teeth eruption starts from 6 months after birth and shedding from 6 years of age [13]. Therefore, perinatal element status is important for teeth structure. Prenatal exposure to toxic metals like lead and manganese have been shown to be associated with adverse birth outcomes and this may affect the morbidity of these children although there is not enough research in this area [14, 15].

Interestingly, higher molar incisor hypomineralisation, a developmental enamel defect, was reported in asthma cases, previously [16, 17]. Despite this, there was no published study in teeth element contents of asthma cases. Simultaneous investigation of blood and dental element levels can contribute to understanding the effect of these elements on the pathogenesis and prognosis of asthma. Here, we aimed to evaluate blood and deciduous tooth element levels (Mg, P, Ca, Cr, Mn, Fe, Cu, Zn, strontium [Sr], Mo, Cd, Pb, Hg) in the bronchial asthma group and the control group. Elemental differences to be detected in dental samples will guide the explanation of the mechanism of the occurrence of asthma cases and will assist in the planning of new studies.

## Methods

A case–control study was conducted at Hacettepe University Faculty of Medicine, between November 2016 and April 2017. Inclusion criteria consisted of cases with well-controlled asthma cases aged 6–12 years without coexistent malnutrition, and any other chronic disorders, and the presence of shed deciduous teeth within the last 3 days. Well-controlled asthma cases were not using any chronic inhaled or oral medication, and they had no symptoms and history of exacerbation within the previous year according to Global Initiative for Asthma (GINA) report [18]. There was no asthma history of their mothers. During the follow-up period, 17 asthma patients donated shed deciduous teeth having neither decay nor filling and enrolled for the study. Following each asthma case, one or two age- (± 1 years) and gender-matched healthy children who gave shed deciduous teeth (n = 26) were recruited for the control group.

The local Ethical Committee approved the protocol. Parents gave verbal and written informed consent after receiving detailed explanations of the study.

Infant (gestational length, birth weight, age, gender of child, breastfeeding duration, vitamin/mineral supplements (yes or no), iron deficiency anemia history (yes or no), oral health (carious teeth and fillings on admission and history of tooth decay), current body weight and height of children), parental characteristics (age, education status, smoking history), and type of donated deciduous teeth (incisors or molar) were recorded in a structured questionnaire (Additional file 1).

The breastfeeding duration of a child was grouped as ≤ 12 and > 12 months. Parental education level was divided as ≤ 8 and > 8 years. The parental smoking status was classified as “both of them”, “only one parent” and “none of them”.

From each enrolled child, teeth were collected in plastic boxes. Venous blood sample (3–4 mL) was taken into ‘metal-free’ tubes containing ethylenediamine tetraacetic acid (BD Vacutainer®) and divided into two parts. One part was studied for hemograms [hemoglobin (Hb), mean corpuscular volume, red cell distribution width, platelets, white blood cell count]. The second part of blood and the teeth samples were stored at + 4°C.

WHO AnthroPlus packet programme estimated “z” scores for height-for-age (HAZ), weight-for-age (WAZ), and body mass index (BAZ) [19].

Inductively coupled plasma mass spectrometry (Agilent Technologies 7700X Series, USA) detected element levels for Fe, Zn, Ca, Mg, P, Sr, Cu, Mn, Mo, Cr, Cd, Pb, and Hg at Certificated Environment Laboratory. Analytic methods for blood and tooth samples and analytic quality control studies are similar to the previous study [10].

### Statistical analysis

The Statistical Package for the Social Sciences (version 22.0, Chicago, IL, USA) performed statistical analyses. The Kolmogorov–Smirnov test and histograms determined the type of distribution. Normal distribution was present in some teeth elements (Mg, P, Ca, and Zn) and some blood elements (Mg, P, Fe, Ca, Cu, Zn, Sr, Mo, Cd). Others showed right-skewed distribution.

The differences between the asthma group and the control group were compared with an independent t-test in normally distributed parameters, and means and standard deviations (SD) were calculated. Mann–Whitney U test was used for the comparison of non-parametric subgroup analysis and quartiles were taken. Frequencies in categorical data were assessed by the Chi-square test and Fischer-exact test when necessary. When a difference was detected in the Chi-square test of the 3 × 2 table, adjusted residuals were calculated for subgroup differences.

For each element level, the generalized linear model investigated differences between the asthma and the control groups. Factors having *p* value less than 0.15 in univariate analysis (child’s BAZ, history of iron deficiency anemia, and status of parental smoking) were taken as covariates. For elements with a normal distribution, identity was taken as the link function. “Gama with log link” was taken for scale response in elements showing right-skewed distribution. Estimated means and 95% Confidence Interval (CI) of elements were calculated.

Statistical tests were two-sided, and statistical significance was at *p* < 0.05.

## Results

The study group consisted of 17 patients with asthma and 26 control cases without any chronic disease. The mean ages and the distribution of gender were similar in the groups (Table 1). The asthma group had a slightly higher BAZ value than the control group (*p* = 0.068). The asthma group and the control group were compared in terms of WAZ, HAZ, gestational length, and maternal age were found to be similar (*p* > 0.05).

**Table 1:** Demographic characteristics of the study population

	Control Group	Asthma Group	*p*
n	26	17	
Child characteristics			
Age, years*	7.8 ± 1.7	7.7 ± 1.8	0.885
Gender, Male, %**	53.8	52.9	0.954
Gestational length, week*	39.1 ± 2.1	38.9 ± 1.7	0.737
Birth weight, gram*	3283 ± 687	3129 ± 383	0.406
Breastfeeding > 12 mo, %**	80.8	87.5	0.690
Formula milk feeding, %**	32.0	23.5	0.731
Fe prophylaxis during infancy period, %**	57.7	47.1	0.494
Iron deficiency anemia history, %**	15.4	35.3	0.158
Vitamin prophylaxis during infancy period, %**	34.6	41.2	0.663
Weight for age, z score^*^	0.17 ± 0.82	0.60 ± 1.33	0.275
Height for age, z score*	0.45 ± 0.93	0.29 ± 1.23	0.628
Body mass index z score^*^	− 0.11 ± 0.95	0.67 ± 1.51	0.068
History of tooth decay, %**	73.1	82.4	0.714
Presence of tooth decay or filling in mouth, %**	46.2	41.2	0.748
Studied tooth samples, incisors, %**	88.5	70.6	0.230
Hemograms			
Hemoglobin, g/dL*	12.8 ± 1.0	12.6 ± 0.8	0.661
Mean corpuscular volume, %*	80.5 ± 5.5	79.7 ± 7.1	0.666
Red cell distribution width, %*	13.7 ± 0.8	14.2 ± 1.2	0.150
White blood cell*, mm^3^	8.3 ± 2.6	8.4 ± 1.7	0.975
Platelets*, mm^3^	304.5 ± 75.6	306.6 ± 86.7	0.933
Parental characteristics			
Maternal age at the birth of index child, years^*^	26.9 ± 6.6	28.3 ± 5.3	0.460
Paternal age at the birth of index child, years^*^	30.9 ± 5.6	32.9 ± 4.9	0.229
Maternal education > 8 years**	65.4	64.7	0.964
Paternal education > 8 years**	75.0	68.8	0.728
Maternal occupation, presence**	61.5	41.2	0.191
Parental smoking**			0.008
None	42.3	11.8^a^	
Only one parent	42.3	29.4^a^	
Both of them	15.4	58.8^b^	

*Mean ± SD; compared with independent t-test

**Data were compared with the Chi-square test and Fischer-exact test when appropriate

^ab^Values having different letter were found to be statistically different, *p* < 0.05

There was no difference between the asthma group and the control group for breastfeeding more than 12 months, the presence of Fe prophylaxis, and vitamin prophylaxis during the infancy period. No difference was present in the frequencies of studied incisor deciduous teeth in groups. The presence of tooth decay of filling in the mouth was 46.2% in the control group and 41.2% in the asthma group (*p* = 0.748). There was no difference in hemogram values in groups (Table 1).

The smoking of both parents was found to be more in the asthma group than those in the control group (*p* = 0.008, Table 1).

### Tooth and blood element levels

Table 2 refers to the levels of elements in the tooth according to groups. The mean tooth Mg level was lower in the asthma group compared with the control group (5.51 ± 1.35 mg/g, 6.41 ± 1.22 mg/g; respectively, *p* = 0.030). On contrary, the mean blood Mg levels were similar among groups (Table 3). The teeth/blood ratio of Mg was lower in the asthma cases than the ratio in the control group (13.6 ± 4.6%, 16.6 ± 4.5%, respectively; *p* = 0.044).

**Table 2:** Concentration of elements within the tooth according to groups, in the asthma group and the control group

	Univariate				GLM^a^		
	Unit	Control	Asthma	*p*	Control	Asthma	*p*
		n = 26	n = 17		n = 26	n = 17	
Mg	mg/g*	6.41 ± 1.22	5.51 ± 1.35	0.030	6.44 [5.91–6.96]	5.46 [4.79–6.14]	0.042
P	mg/g*	139 ± 20	132 ± 26	0.378	139 [130–149]	131 [119–143]	0.332
Mg/P	% ratio*	4.66 ± 0.94	4.30 ± 1.19	0.283	4.67 [4.24–5.10]	4.29 [3.73–4.85]	0.336
Ca	mg/g*	244 ± 44	229 ± 56	0.353	249 [229–269]	222 [196–247]	0.140
Cr	µg/g**	0.08 (0.04; 0.18)	0.07 (0.05; 0.17)	0.842	0.13 [0.09–0.18]	0.11 [0.07–0.17]	0.585
Mn	µg/g**	1.15 (0.77; 1.72)	1.09 (0.47; 1.54)	0.441	1.29 [1.01–1.64]	1.04 [0.76–1.41]	0.305
Fe	µg/g**	5.56 (3.88; 9.43)	6.53 (2.99; 8.64)	0.785	8.26 [6.16–11.07]	6.84 [4.69–10.00]	0.478
Zn	µg/g*	120 ± 27	111 ± 23	0.244	120 [110–130]	111 [98–124]	0.303
Cu	µg/g**	0.22 (0.12; 0.45)	0.30 (0.16; 0.76)	0.345	0.48 [0.32–0.74]	0.53 [0.31–0.92]	0.796
Zn/Cu	Ratio**	553 (229; 1023)	364 (148; 714)	0.285	786 [533–1160]	413 [247–690]	0.084
Sr	µg/g**	61 (50;104)	73 (50; 82)	0.901	82 [66–103]	91 [68–122]	0.613
Mo	µg/g**	0.02 (0.02; 0.04)	0.02 (0.01; 0.05)	0.901	0.04 [0.02–0.05]	0.05 [0.03–0.09]	0.397
Cd	≥ DL***	69.2%	88.2%	0.149			
Pb	µg/g**	0.59 (0.50; 0.76)	0.67 (0.45; 0.91)	0.619	0.70 [0.59–0.83]	0.68 [0.55–0.85]	0.889
Hg	≥ DL***	46.2%	29.4%	0.272			

*Mean ± SD; compared with independent t-test

**Median (25; 75p); compared with Mann–Whitney U test

***%; compared with the Chi-square test and Fischer-exact test when appropriate

^a^Estimated marginal mean [95% Confidence Interval]; adjusted for child’s BAZ, history of iron deficiency anemia, and status of parental smoking

**Table 3:** Concentration of elements within the blood according to groups, in the asthma group and the control group

	Univariate				GLM^a^		
	Unit	Control	Asthma	*p*	Control	Asthma	*p*
		n = 26	n = 17		n = 26	n = 17	
Mg	mg/L*	39.9 ± 6.8	41.7 ± 5.7	0.385	39.8 [37.3–42.3]	41.9 [38.6–45.2]	0.365
P	mg/L*	402 ± 69	408 ± 48	0.786	399 [374–425]	413 [379–446]	0.570
Mg/P	%ratio**	9.3 (8.8; 11.8)	10.0 (8.7; 11.0)	0.709	10.1 [9.4–10.9]	10.3 [9.4–11.3]	0.811
Ca	mg/L*	64.8 ± 7.5	65.1 ± 6.7	0.894	65.0 [62.1–68.0]	64.8 [61.0–68.6]	0.936
Cr	µg/L**	0.81 (0.76; 0.94)	0.82 (0.77; 0.95)	0.535	0.85 [0.77–0.92]	0.89 [0.79–0.99]	0.575
Mn	µg/L**	9.6 (8.3; 13.4)	9.0 (7.6; 11.2)	0.214	10.7 [9.5–11.9]	9.5 [8.2–11.0]	0.272
Fe	mg/L*	467 ± 66	467 ± 44	0.968	466 [444–488]	468 [439–497]	0.932
Zn	mg/L*	5.86 ± 1.16	5.04 ± 1.06	0.026	5.89 [5.43–6.36]	4.99 [4.38–5.59]	0.034
Cu	mg/L*	1.05 ± 0.17	1.13 ± 0.19	0.182	1.05 [0.98–1.13]	1.12 [1.02–1.21]	0.349
Zn/Cu	Ratio*	5.64 ± 1.03	4.54 ± 0.92	0.001	5.66 [5.25–6.07]	4.50 [3.97–5.03]	0.002
Sr	µg/L*	18.6 ± 5.8	17.9 ± 7.4	0.757	17.2 [14.8–19.7]	19.9 [16.7–23.1]	0.237
Mo	µg/L*	0.93 ± 0.38	0.86 ± 0.33	0.514	0.96 [0.82–1.10]	0.81 [0.63–1.00]	0.264
Cd	µg/L**	0.16 (0.09; 0.23)	0.15 (0.12; 0.22)	0.842	0.18 [0.14–0.21]	0.15 [0.12–0.20]	0.425
Pb	µg/L**	9.5 (7.3; 12.5)	9.9 (8.5; 12.0)	0.502	11.2 [9.5–13.1]	9.9 [8.0–12.1]	0.386
Hg	µg/L**	0.18 (0.10; 0.36)	0.20 (0.15; 0.28)	0.365	0.29 [0.20–0.41]	0.24 [0.15–0.38]	0.559

*Mean ± SD; compared with independent t-test

**Median (25; 75p); compared with Mann–Whitney U test

***%; compared with the Chi-square test and Fischer-exact test when appropriate

^a^Estimated marginal mean [95% Confidence Interval]; adjusted for child’s BAZ, history of iron deficiency anemia, and status of parental smoking

Tooth Zn levels were similar in groups (*p* = 0.244, Table 2). The mean blood Zn level was 5.04 ± 1.06 mg/L in the asthma group and 5.86 ± 1.16 mg/L in the control group, which was also detected to be statistically significant (*p* = 0.026, Table 3). The blood Zn/Cu ratio was also lower in the asthma cases than the ratio in the control group (4.54 ± 0.92, 5.64 ± 1.03; respectively, *p* = 0.001, Table 2). The teeth/blood ratio of Zn was similar in the asthma cases and the control group (22.4 ± 4.6, 21.4 ± 6.8, respectively; *p* = 0.592).

No significant differences were detected in P, Ca, Cr, Mn, Fe, Cu, Sr, Mo, Cd, Pb, Hg levels in tooth and blood samples (Tables 2 and 3).

### Multivariate analysis

When child’s BAZ, history of iron deficiency anemia, and status of parental smoking were controlled, the generalized linear model detected lower tooth Mg levels in the asthma group compared to the control group (mean [95% CI]: 5.46 [4.79–6.14], 6.44 [5.91–6.96] mg/g, respectively, *p* = 0.042). Asthma and the control group had similar teeth P levels and teeth Mg/P ratios (Table 2).

After adjusting the child’s BAZ, history of iron deficiency anemia, and status of parental smoking, the generalized linear model revealed significantly lower blood Zn levels and Zn/Cu ratio in asthma cases than the control group (mean [95% CI]: 4.99 [4.38–5.59], 5.89 [5.43–6.36] mg/L, respectively; *p* = 0.034 for blood Zn levels and 4.50 [3.97–5.03], 5.66 [5.25–6.07], respectively; *p* = 0.002 for Zn/Cu ratio). However, blood Cu levels were similar in groups (Table 3).

Asthma cases had slightly lower tooth/blood ratio of Mg than the control group after controling for confounding factors (mean [95% CI]: 13.5 [11.2–15.8], 16.6 [14.9–15.8], respectively; *p* = 0.056). However, tooth/blood Zn levels did not differ in asthma and control group (mean [95% CI]: 21.2 [19.0–23.5], 22.7 [19.7–26.1], respectively; *p* = 0.480).

## Discussion

Some element levels from two biological samples, including blood Zn and tooth Mg were found to differ in asthma cases in our study.

The asthma group had significantly lower blood Zn values. Interestingly, a previous meta-analysis revealed that higher maternal Zn intake during the pregnancy period was associated with lower odds of wheeze during childhood [20]. In a recent study by Pan et al. it was concluded that Zn deficiency may be related to the IgE production and this relationship would increase the risk of asthma [21]. Dysregulation of Zn homeostasis was also proposed to reduce antioxidant functions and increase the risk of asthma development [22–24]. Despite this, the association between serum Zn levels and asthma pathogenesis remains controversial; some studies reported to have no significant association [2, 23–25], others showed low levels [22, 26, 27] or high levels [27]. Oeffelen et al. did not detect any association between serum Zn concentrations and childhood asthma in the PIAMA birth cohort study [25]. In the meantime, the serum Zn levels in the bronchial asthma group were found to be higher only in adult females than the control group; however, a positive correlation was reported between serum Zn levels and serum opsonic activity in both genders [27]. Differences might be explained by studied biologic samples, methods, age groups, and disease severity. Furthermore, a meta-analysis including twenty-six studies suggested that the asthma group had lower circulating Zn levels compared with the control groups [8]. Further studies with different biologic matrixes will clarify the situation.

Low Mg levels were seen in the tooth of asthma patients in this study. Low teeth Mg levels might propose the importance of perinatal Mg status in asthma pathogenesis, starting from the intrauterine period and infancy. As known, Mg causes the relaxation of bronchial smooth muscles. Reduced intake of Mg induces the worsening of pulmonary functions [28, 29]. In addition, there was tooth hypomineralisation in asthma cases [17]. Interestingly, Liu et al. showed Mg could be an effective inducer of osteoblastic differentiation of stem cells from exfoliated deciduous teeth [30]. On the other hand, the blood Mg levels did not differ in asthma cases from our study. In addition, a meta-analysis of 34 studies showed that asthma cases had a lower level of Mg than the control group in the Asian population [2]. Alsharnoubi et al. also reported that Mg deficiency could lead to asthma development in children [31]. Another study reported a consistent inverse non-significant association between serum Mg levels and asthma prevalence in children at 8 years of age [25]. These differences could be due to the studied age of patients, disease severity, given medications, Mg homeostasis, studied biologic matrix such as blood, serum, teeth.

Blood and teeth Hg, Pb, and Cd levels were similar in the astma group and the control group in our study. This can be explained by cases having no known history of toxic metal contact in his or her family. Heinrich et al. found no differences in Hg levels in both blood and urine samples for asthma, wheezing, bronchial hyperresponsiveness, and allergic sensitization in children (n = 1056) aged 5–14 years from low exposure cases [32]. No influence of Hg levels on asthma was also stated in Korean adults [33] and a cross-sectional study with 5866 children aged 2–15 years from the NHANES survey 2007–2012 [34]. Similar to our study, Wells et al. reported similar blood Pb (1.13 μg/dL) levels in a cross-sectional study of 1788 children and no association with asthma, atopic asthma, or general atopy, however, an increase in blood Pb [35] resulted in a significant increase in IgE and eosinophils [11.1% (95% CI 5.6, 16.9) and 4.9% (2.3, 7.6) per 10 μg/L Pb]. In addition, Rabito et al. showed no association between blood Pb levels of children aged 18–24 months and asthma diagnosis at 3 and 9 years of age [36]. On the other hand, inconsistent publications were also present. Wang et al. described a positive association for basal blood Pb with asthma in kindergarten children from Taiwan (n = 930). However, interaction with total serum IgE only in boys [37]. Wu et al. noted that cases with higher blood Pb levels had a higher odds for active asthma (OR = 1.24), wheezing or whistling (OR = 1.19) in the 6–11 age group in a cross-sectional study [34]. No significant association in adult cases aged 20–79 years was reported between serum Pb with current wheeze or current asthma, regardless of smoking status [38]. Park et al. reported that the highest quintile of blood Pb revealed 67% higher odds for asthma compared to the lowest quintile in Korean adults [33]. Similarly, the most top quintile of blood Cd resulted in 55% more risks than the lowest quintile [33]. Yang et al. reported a significant association for serum Cd with lower percentages of FEV1 and FEV1/FVC predicted in all adult participants and with current asthma in smokers [38]. On the other hand, environmental tobacco smoke is a common risk factor for exposure to numerous toxic substances and also wheezing and asthma in children [3, 4]. Given the higher frequency in asthma cases in our study, both maternal (slightly higher) and paternal smoke exposure (significantly higher) should be discussed in prenatal and perinatal developmental origins of childhood asthma.

No variations between the asthma group and healthy controls were detected in elements including Ca, Fe, Cu, P, Cr, Mn, Sr, and Mo levels in our study. A meta-analysis showed that asthma cases had markedly higher levels of Cu and Fe among overall populations and Asians [2]. A previous study also reported that the serum Cu concentrations in bronchial asthma patients tended to be higher compared with healthy individuals [24]. Higher levels of Cu and Fe may induce oxidative stress and chronic inflammation in asthma cases [2]. A study including controlled asthma, uncontrolled asthma, and control group investigated, Cu and Pb levels were increased in asthmatic groups and these levels were increased more obviously in moderate cases compared with the mild group [31]. Only one case–control study has investigated the role of Mn in asthma, and they concluded that Mn dietary intake is inversely related to bronchial hyperreactivity [24]. We have also measured the levels of Mn in both tooth and blood matrixes. Although the tooth and blood Mn levels were low in asthma patients, it was not statistically significant. Further studies with a large sample size could explain this relation.

Our results also showed that asthma cases had significantly higher parental smoke exposure and smoking history. This finding once again demonstrated to us the effect of smoke exposure on the pathogenesis of asthma as reported previously [39].

Some limitations were present in this study. The small sample size decreases the power of the study. Also, due to the cross-sectional design of this study, an accurate cause-effect relationship could not be formed. However, there are some strengths in the study. It is not easy to collect healthy deciduous teeth as a biological sample. Teeth samples may show perinatal exposure of children and could explain the pathogenesis of diseases [12]. This is the first study evaluating element levels in deciduous teeth from asthma cases. In addition, previous studies explored one or three elements in only one biological sample. However, in our study, several blood elements (n = 13) were analyzed in two separate biological materials at the same time. This study detecting the difference in the tooth sample will guide further studies evaluating asthma mechanisms.

## Conclusion

Our study revealed low tooth Mg levels and low blood Zn levels in the asthma group. Further studies on the zinc and magnesium levels of asthma at different severity levels will contribute to the interpretation of the results of the current study. Mg and Zn status could be evaluated in future studies in the management of asthma cases. Our study will shed light on other studies about the management of asthma cases. Further studies are necessary to show the relationship between asthma and elements for understanding asthma development and the prevention of disease.

## Supplementary Information

**Additional file 1**. Data collection form.

## Data Availability

Research data can be requested from the corresponding author.
