# Supplementary material for: Evaluation of blood and tooth element status in asthma cases: a preliminary case–control study
Source: BMC Pulm Med. 2021 Jun 15;21:201. doi: 10.1186/s12890-021-01565-9 (PMC8204585; doi:10.1186/s12890-021-01565-9)
Supplement: Supplementary file 1 — Additional file 1. Data collection form. [file 12890_2021_1565_MOESM1_ESM.docx]

**EVALUATİON OF BLOOD AND TOOTH ELEMENT STATUS İN ASTHMA CASES:**

**A PRELIMINARY CASE-CONTROL STUDY**

**S. Songül YALÇIN, Nagehan EMİRALİOĞLU, Suzan YALÇIN**

**Case No: ……..**

| **Parental characteristics** | **Mother** | **Father** |
| --- | --- | --- |
| Age | ……..years | ……..years |
| Education duration | a)Primary b)Secondary  c)High school d)University | a)Primary b)Secondary  c)High school d)University |
| Have a job | a)yes b)no | a)yes b)no |
| Smoking status | a)smoking  b)environmental smoke exposure  c) neither A nor B | a)smoking  b)environmental smoke exposure  c) neither A nor B |
| Weight/ Height | ….….kg/ …….cm | ….….kg/ …….cm |

**Child’s characteristics**

| Please state presence of any disease under physician control | ………………… |
| --- | --- |
| Is your child use any drug prescribed by a physician? | a)yes ………… b)no |
| Child Age | …………..years* |
| Gender | (a) male (b) female |
| Gestational length | ………… week |
| Birth weight | ………… gram |
| Total duration of breastfeeding | ………….mo |
| Did you give any infant formula to your child? | a)yes b)no |
| Did you give any Fe prophylaxis during infancy period? | a)yes b)no |
| Did your child have iron deficiency anemia | a)yes b)no |
| Did you give any vitamin prophylaxis during infancy period? | a)yes b)no |
| Did your child have any history of tooth decay? | a)yes (no:..) b)no |
| Did your child have history of tooth filling? | a)yes (no:..) b)no |
| Type of donated deciduous teeth | (a) incisors (b) molar |
| Is there any filing or decay in donated deciduous teeth ? | a)yes b)no |
| Examinations |  |
| Weight/ Height | ….….kg/ …….cm |
| Hemoglobin | ….,,,.g/dL |
| Mean corpuscular volume | …,…% |
| Red cell distribution width | ...…..% |
| White blood cell | …… mm^3^ |
| Platelets | ……..mm^3^ |
